# Recurrent Nipah outbreaks in Kerala: implications for health policy and preparedness

**DOI:** 10.3389/fpubh.2024.1356515

**Published:** 2024-06-20

**Authors:** Asuma Ayesha Rahim, Priya Chandran, V. Bindu, Chandini Radhakrishnan, Anitha P. Moorkoth, Lathika Velichapat Ramakrishnan

**Affiliations:** ^1^Department of Community Medicine, Government Medical College Kozhikode, Kozhikode, India; ^2^Department of Emergency Medicine, Government Medical College Kozhikode, Kozhikode, India; ^3^Department of Microbiology, Government Medical College Kozhikode, Kozhikode, India; ^4^District Medical Office (DMO-H), Kozhikode, India

**Keywords:** Nipah infection, NiV, outbreak, Kerala, recurrence, policy implications

## Summary

Nipah virus (NiV) infection, a highly pathogenic emerging zoonotic disease associated with significant mortality rates, is prevalent in South East Asian countries. This infection typically manifests in small clusters, predominantly presenting as either encephalitis or acute respiratory distress. In India, NiV has been documented in the states of West Bengal and Kerala, with four of the six reported outbreaks occurring in Kerala. This study focuses on the epidemiology of NiV infection in Kerala, offering insights and implications for future policies.

Epidemiologically, three of the four outbreaks in Kerala occurred in a specific geographic belt, suggesting a consistent factor in the spillover of infection from reservoir bats. The average age of affected individuals was 41 years, with a male predominance. The mean incubation period was determined to be 9 days, and transmission primarily occurred within healthcare settings due to lapses in infection prevention and control practices. Intensive response strategies including case isolation, contact tracing, and surveillance were consistently employed during all outbreaks. Challenges related to diagnosis and treatment were addressed through the development and regular updates of state guidelines for outbreak response.

The article emphasizes the need for fortifying the state's health system to enhance preparedness for future outbreaks. This involves proactive measures such as improving infection control practices, expediting diagnostic processes, and exploring advanced treatment options. Strengthening the surveillance system, particularly for monitoring acute encephalitis syndrome (AES) and acute respiratory distress syndrome (ARDS) is crucial for the early detection and containment of potential outbreaks and thereby mitigate the impact of future outbreaks in the region.

## Background

India witnessed its sixth outbreak of Nipah virus infection in September 2023, in the southern state of Kerala when the Nipah virus (NiV) was isolated from two patients with epidemiological link to a probable case of NiV infection. Encephalitis caused by NiV is an emerging infectious disease of public health importance reported from South-East Asian countries. Both animal-to-human and human-to-human transmission have been documented (1). Between 1998 and 2015, over 630 cases of NiV infections were reported in Malaysia, Singapore, Bangladesh and India (2).

While the Malaysian outbreak reported 276 cases with a 38% fatality rate, subsequent outbreaks in India and Bangladesh witnessed significantly higher case fatality rates ranging from 43% to 100% (3). Fruit bats of the genus Pteropus serve as the natural reservoir for the virus and the virus has been isolated from bat urine and partially eaten fruits in Malaysia (4). Human infection typically begin with fever and brain inflammation leading to disorientation or coma (5). Some patients also present with acute respiratory distress syndrome (ARDS). Laboratory confirmation is done by Serum Neutralization antibody detection, Enzyme linked immune sorbent assay (ELISA), or Real time reverse transcriptase polymerase chain reaction (RT-PCR) tests. NiV needs to be handled in a Bio-safety level 4 containment facility and most countries in South East Asia lack diagnostic facilities.

Out of the six outbreaks in India since 2001, four have occurred in Kerala, with three in the Northern district of Kozhikode. The authors being part of the Nipah outbreak response team have been actively involved in the containment of all the three outbreaks in the region. This recurring phenomenon in the district of Kozhikode over the last 5 years raises questions about transmission dynamics.

The authors conducted an analysis of epidemiology, clinical presentations and health system responses of all four outbreaks in Kerala to identify gaps in our current knowledge of Nipah infection epidemiology and its potential policy implications.

## The first outbreak-2018

The initial outbreak caught the health system off guard, with limited experience and knowledge to fight the virus. The outbreak was identified due to clustering of cases within a household and later in three health care institutions. Of the 18 cases confirmed by RT-PCR, 16 succumbed (case fatality rate - 88.8%). In addition there were four probable cases (not lab confirmed) identified retrospectively though audits of deaths due to AES & ARDS with symptoms suggestive of NiV infection who expired prior to confirmation of the outbreak. The mean incubation period was 9 days and mean age of the affected was 41 years with male preponderance.

The Primary case, identified retrospectively in a tertiary hospital served as a point source for 15 other cases including two health care workers. The transmission occurred person to person, mainly in health care settings. More than 2,600 contacts were under surveillance for a period of 21 days (maximum incubation period) including 239 contacts from Malappuram district (6). Department of Community medicine at the Government Medical colleges in both districts took the lead in contact tracing and surveillance in collaboration with the District health system. The basic reproduction number (R_0_) from May 20th for the ensuing 4 week period was calculated as 0.4, which indicated the epidemic to be dying out. The outbreak was contained and declared over on 10th June 2018 (7).

The source of infection for the primary case could not be identified and was assumed to be contact with fruit bats from the forest or consumption of fruits contaminated with bat secretions. No other animal reservoir could be identified as an intermediate host for spill over infection to humans. A coordinated rapid outbreak response by the health system led to the containment of the outbreak within 3 weeks and was declared closed by July the same year. The authors evaluated district and state coordinated actions using the Management science for health frame work tool (MSH framework) (7).

## The second outbreak−2019

In June 2019 Nipah revisited Kerala, this time in Ernakulam district. A 21 year old male student presented with fever and signs of encephalitis. NiV infection was confirmed by RT-PCR of throat swab, urine and serum samples at National Institute of Virology (NIV) Pune (8). Equipped with the 2018 experience and with the management and control guidelines against NiV infection in place, this time no further spread occurred and the patient recovered. The source of infection in this instance also could not be confirmed conclusively and was presumed to be due to consumption of fruits contaminated with bat secretions. Robust contact tracing involving 330 contacts by the district surveillance team categorization of risk using an algorithm developed in the state into high and low risk contributed to effective containment. High risk individuals included those with direct contact with body fluids of confirmed or probable cases or sharing a closed space for more than 12 h with a confirmed case (9). An onsite field laboratory was also set up for conducting Point of care test, RTPCR and ELISA.

## The third outbreak in the midst of the COVID-19 pandemic

Nipah hit Kozhikode again in September 2021 while the state was experiencing a second wave of the COVID-19 pandemic (10). A 12 year old boy presented with fever progressing to encephalitis within a period of 5 days. He had sought treatment from three health care facilities prior to confirmation of diagnosis and succumbing to the disease. Contact tracing identified 240 contacts but no further cases were identified. Keeping in view the ongoing COVID-19 pandemic all contacts were also screened for SARS-CoV-2 (12 were positive).

Source of infection in this case was also presumed to be consumption of contaminated fruits from the orchard of exotic fruits owned by the family. Bat studies conducted in the area showed presence of NiV antibodies but viral RNA could not be detected in the bats. We speculate that stringent COVID-19 infection control measures, universal masking at health care facilities and among the public, played a crucial role in limiting the outbreak to a single case.

## The fourth outbreak−2023

The most recent outbreak in Kozhikode district in September 2023 reported six confirmed cases and two fatalities. Index case was a 9 year old child who presented with fever rapidly progressing to encephalitis in 5 days. Similar to the first outbreak, the primary case (father of the child) was identified retrospectively and succumbed in a private health care facility transmitting infection to five others, including one health care worker. Fever was the predominant symptom and contrary to prior outbreaks, respiratory distress was the common presentation (with the exception of a single case of encephalitis). Male gender and mean incubation period of 9 days were consistent with the initial outbreak.

## Epidemiological analysis

All four events were considered as outbreaks even though only one case was involved in 2019 and 2021 as NiV is a rare pathogen and moreover the disease was new to the community (11, 12). From an epidemiologists perspective male preponderance in infections potentially linked to engagement in outdoor activities as revealed through in-depth field based case investigations. All the four outbreaks pooled together, lowest age was 9 years with majority between the age of 30–45 years (6, 8, 10). All three spill overs in Kozhikode district occurred in the same geographic belt, suggesting a common link possibly environmental, behavioral or a combination of both. The initial spill overs occurred during May- June coinciding with the breeding season for the Pteropus bats, contrast with later outbreaks in September, raising the possibility of spill over incidents all year through (13). This is in contrast to outbreaks in South East Asia which have occurred during the classic NiV transmission season (December–May) (3).

The affected geographic areas in Kozhikode share common features, including the presence of plantation areas with arecanut and fruit trees, including exotic fruits, adjacent to a natural forest cover of 300 acres which is home to several bat species. Initial epidemiological investigations conducted during outbreaks in these areas revealed presence of large number of fruit bats, with half eaten fruits abundantly found at the outbreak sites. Recent observations during field visits and environmental surveys conducted by the team in the present outbreak point to the primary case's habit of plucking fruits in the peri-domestic area and from his plantations. Multiple fruit trees surround the area, and the primary cases house was ~4–5 km from the forest belt of Janakikkadu.

Mapping of the bat population and testing for presence of NiV among the bats in all outbreaks by National Institute of Virology (NIV) showed that 19% of the P medius were positive for NiV and Anti NiV antibody in 2018 (14) while 21% of P medius and 37.7% of Rousettus leschenautica exhibited anti NiV Antibody in 2019 outbreak (10). Anti NiV antibodies were demonstrated in bats from the affected area during 2021, confirming bats as the reservoir (10). However the same could not be demonstrated in 2023 from the samples of bats, animal droppings, and half-eaten fruits collected from the village where the initial cases resided (15). NiV human sequences from Kozhikode outbreak and NiV sequences from bat study have shown that the NiV strain circulating in south India are distinct from the Bangladesh strain and a separate “Indian (I) strain has been hypothesized for South India (15).

Considering transmission dynamics, although respiratory secretions and body fluids have been implicated in transmission, gaps in the high risk behaviors predisposing to infection remain unclear. Person to person transmission was established in the 2018 and 2023 outbreak (6) occurring mainly in health care settings indicating that spread occurred in the late symptomatic stage of the disease (6). Most of the secondary cases reported close contact with the primary case. Risk factors for transmission included feeding, contact with body fluids, close contact with a Nipah patient during caregiving and sharing room/space in the hospital (10).

Fever appears to be the predominant symptom in all cases, while encephalitic symptoms like disorientation and seizures were observed more in the 2018 outbreak, the symptoms of respiratory distress were more predominant in the 2023 outbreak. The multiplicity of symptoms pose challenges in early detection by the surveillance system which has to rely on clustering of cases or unexplained deaths as indicators of possible outbreaks. In the 2023 outbreak the survivors were treated with antivirals Remdesvir and Favipiravir, and one case required intensive ventilation. The early initiation of treatment and aggressive supportive measures may account for the low case fatality in the current outbreak. Ribavarin given to a subset of patients in 2018 showed a decrease in encephalitis caused by NiV though the results were not statistically significant (16).

## Challenges and policy implications

Exploring the reasons behind the recurring spill over events of NiV infection leading to outbreaks in Kerala, especially in Kozhikode should be prioritized. Unlike Bangladesh where consumption of raw date palm sap is implicated in transmission (17), the source of infection for the primary case and the mechanism of spill over from the reservoir bats remains unestablished in all the outbreaks in the state. Ingestion of fruits coming in contact with saliva or inhalation of tiny droplets produced from infected urine or saliva of bats roosting among braches of trees can be an important mode of transmission of NiV infection to humans (18). Demonstration of the agent in fruit samples remains elusive hinting at viability of the virus outside the reservoir species. This missing link needs to be explored aggressively to complete the natural history of NiV infection.

*Outbreak response:* Outbreak response in all the instances have been intensive and prompt. Isolation of cases, triaging, contact tracing, risk stratification and surveillance as the basis of containment strategies. Identifying a suitable isolation area and implementing standard operating protocols for isolation and triaging was challenging in earlier outbreaks. Coordination between various sectors viz animal husbandry, health services, medical college, private sector, law enforcement, agriculture and central agencies (NIV, ICMR, NCDC, NIE) was crucial for successful containment. Tracing of community contacts was always a challenge considering the multiple health care institutions visited by the cases and participation in social events (funerals, prayers etc.). The number of health care workers stratified as high risk contacts was of concern indicating lapses in adherence to infection control practices even in the recent outbreak. Hospitals should enforce policies mandating face masks for Health care workers and care-givers, especially in the Emergency and Intensive care settings. In the recent outbreak a Cluster Containment Strategy was undertaken by the district administration by declaring seven village panchayats as containment zones adapting the containment plan guideline for SARS-CoV-2 (19).

*Confirmation of diagnosis:* Confirmation of diagnosis posed a challenge in the initial outbreak, requiring sample transportation to NIV Pune. This challenge was addressed to a great extent in 2019 with the introduction of point of care (POC) micro PCR assay at designated tertiary care institutions. POC test proved to be a game changer in the outbreak response activities in the subsequent outbreaks as delay in diagnosis could be mitigated to a great extent but confirmation still necessitated a Biosafety Level 3 (BSL3) facility. Establishment of BSL 3 lab in the region is crucial for future outbreak preparedness.

*Treatment protocols:* There are no drugs or vaccines specific for Nipah virus infection recommended by the WHO or CDC even though WHO has identified NiV infection as a priority disease for research and development (20, 21). The absence of a protocol for management of the outbreak was addressed as early as 2018 with guidelines for contact tracing, surveillance and treatment guidelines framed by the Government of Kerala with contributions from experts from National agencies, and revised in subsequent outbreaks (22). The guidelines should be reviewed based on newer evidences. The early initiation of Remdesvir in the in the latest outbreak showed promising results (23). Its efficacy as a drug for post exposure prophylaxis needs to be explored further (22). Monoclonal antibody (m 102.4) treatment was considered in the first outbreak and Standard operating procedures for administration were developed by the Indian Council of Medical research (ICMR). Considering the fact that it was still an experimental treatment, medical board of the institution along with central agencies (ICMR) decided to make it available for the patients on compassionate grounds. But m 102.4 was not used as in all instances the outbreak was contained quickly and the opportunity for its use did not arise. Research addressing the development of monoclonal antibodies for future use is the need of the hour and the Government of Kerala has taken the initiative of collaborating with central agencies for development of an indigenous monoclonal antibody which may be of use in future outbreaks.

Contact tracing and surveillance in all four outbreaks was resource intensive (more than 2600 contacts in 2018 and 1260 contacts were traced in the 2023 outbreak). Risk stratification as high risk and low risk contacts were based on treatment and surveillance guidelines of Kerala state (20). Risk stratification needs to be updated on the basis of evidence on exposure risks calculated from all four outbreaks. Aggressive contact tracing with improper risk stratification over burdens the health system causing unnecessary panic in the community. Restrictions imposed such as containment zones can lead to adverse economic impact and the suitability of this approach in curtailing Nipah outbreaks needs to be reviewed.

## Conclusion and recommendation

Preparedness is a key to containment of any outbreak of infectious origin. Early detection is crucial in containing the spread of the virus and mitigating fatality rates. An Event based surveillance mechanism to detect signals or clusters from hospitals/ community is necessary in light of recurring outbreaks. A public private partnership for surveillance can aid early case detection and response. Strengthening of Surveillance of AES and ARDS in the state and subjecting unclassified AES & ARDS to POC test will augment early detection. Reinforcing infection prevention and control practices among health care workers will have broader implications for curtailing other infectious pathogens as well.

Advocating a one health approach with inter sectoral coordination is crucial to address gaps in the natural history of the disease. Exploring alternate routes of spill over and continuing surveillance of NiV among bat population will aid in predicting the risk of potential outbreaks.

Trials with specific antivirals and monoclonal antibodies, research on innovative vaccines and immunological are imperative for evidence based treatment and reducing mortality rates. The political will exhibited by the government in addressing previous outbreaks is commendable and evidence backed policy changes will go a long way in preparing for future challenges.

## Author contributions

AR: Conceptualization, Investigation, Methodology, Project administration, Resources, Supervision, Writing – review & editing. PC: Conceptualization, Formal analysis, Investigation, Methodology, Resources, Supervision, Writing – original draft, Writing – review & editing. VB: Data curation, Investigation, Project administration, Resources, Writing – review & editing. CR: Conceptualization, Resources, Supervision, Validation, Writing – review & editing. AM: Data curation, Investigation, Project administration, Resources, Supervision, Writing – review & editing. LR: Investigation, Methodology, Project administration, Resources, Supervision, Writing – review & editing.
